# Chronopharmacology of AAV gene therapy in mice

**DOI:** 10.17912/micropub.biology.001683

**Published:** 2025-11-12

**Authors:** Patrick Erickson, Alexandra Burr, Biju Parekkadan

**Affiliations:** 1 Chemical and Biochemical Engineering, Rutgers, The State University of New Jersey, New Brunswick, New Jersey, United States; 2 Biomedical Engineering, Rutgers, The State University of New Jersey, New Brunswick, New Jersey, United States

## Abstract

The promise of adeno-associated virus (AAV) vectors for gene therapy is held back by the cost and toxicity of the large doses required. This experiment explored the chronopharmacology of AAVs in mice by studying how circadian phase at the time of injection impacted AAV efficacy and revealed that intraperitoneal doses of AAVs injected during the resting phase (ZT6, 12:00PM) produced greater transgene expression over several weeks than equivalent doses injected during the waking phase (ZT18, 12:00AM). This insight could lead to future work that improves safety and reduces costs of AAV gene therapy and other nanoparticle therapies.

**Figure 1. Transgene expression following AAV injection during the waking or resting circadian phase:**
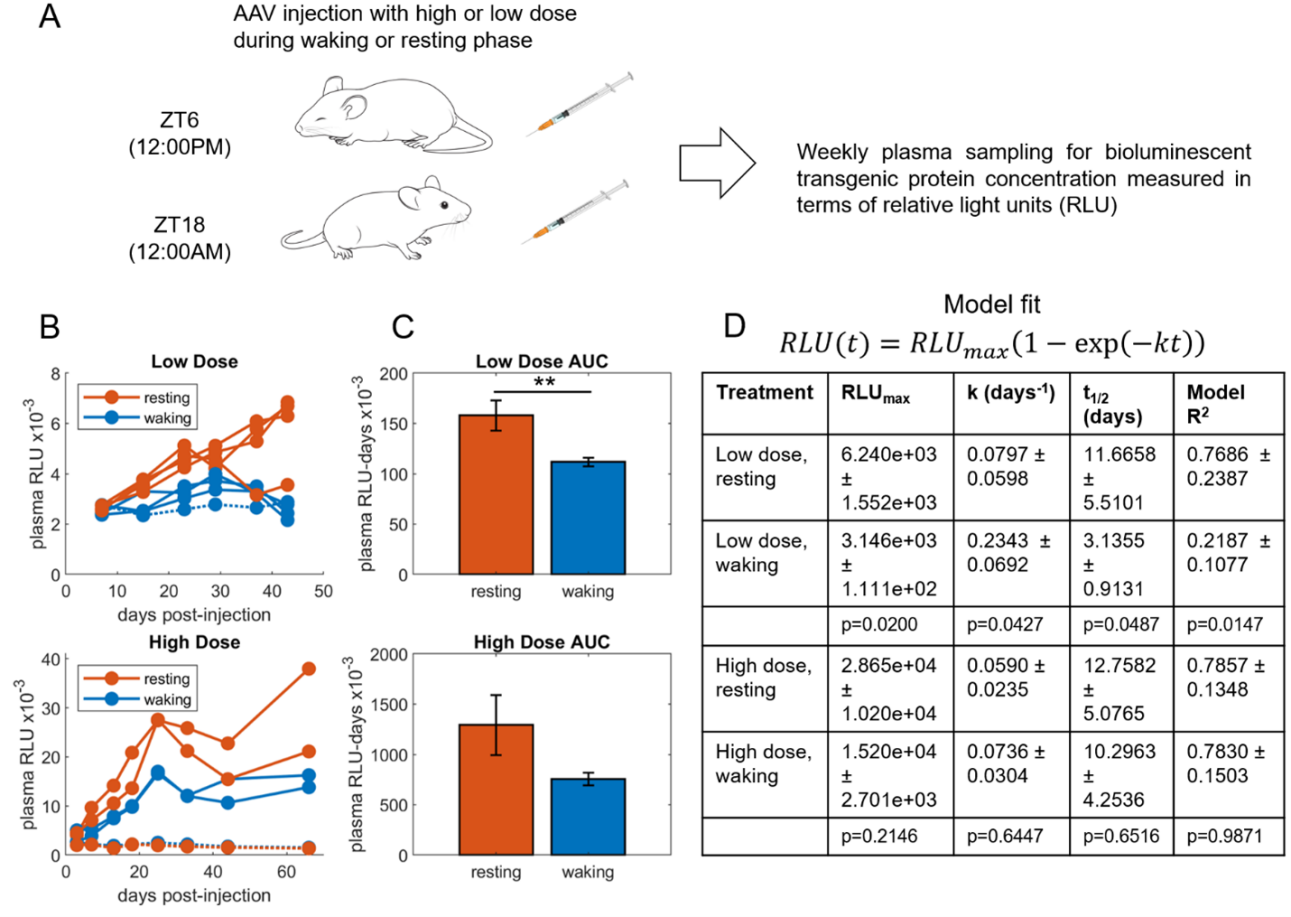
(A) Each mouse received an intraperitoneal injection of AAV either during its Resting phase (ZT6, 12:00PM) or its Waking phase (ZT18, 12:00AM) and plasma levels of the transgenic protein *Gaussia* luciferase were measured in terms of relative light units (RLU) each week. (B) The Low Dose groups received 2.5x10 ^9^ viral genomes per mouse (n=4 per group), and the RLU of the Resting group steadily rose above the Waking group. The High Dose group received 1.0x10 ^10^ viral genomes per mouse (n=3 per group), and again, the RLU of the Resting group individuals were higher than the Waking group. Three mice were found to be non-responders, defined as mice whose data could not be fit by the model in panel D, indicating no transduction, and were excluded from the statistical analyses in the figure. (C) Areas under the curve (AUCs) were compared between Waking and Resting groups for each dose using a two-tailed t test, revealing a statistically significant difference between the Low Dose groups (p=0.0037, indicated by **), but not between the High Dose groups (p=0.1311). (D) Previous work demonstrated that the equation shown is an interpretable mechanistic model of expression dynamics and fits the RLU vs. time data well. The equation was fit to the data of each individual mouse of the four treatment groups. Fit parameters include RLU _max_ (the RLU approached by the curves as time goes to infinity) and k, the rate constant. The time required for the curve to reach half of RLU _max_ was calculated as t _1/2_ , and R ^2^ is the model fit. The mean ± standard deviation of the individuals in each group are shown, along with p-values of t-tests comparing corresponding Resting and Waking groups. Repeating the statistical analyses without excluding the non-responders yields the following for the Low Dose (Resting/Waking/p-value): AUC 1.579e+05 ± 1.484e+04/1.074e+05 ± 9.256e+03/p=0.0012; RLU _max_ 6.240e+03 ± 1.553e+03/3.026e+03 ± 2.569e+03/p=0.0064; k 0.0797 ± 0.0598/1.6121 ± 2.7561/p=0.3008; t _1/2_ 11.6658 ± 5.5101/2.3818 ± 1.6817/p=0.0181; R ^2^ 0.7686 ± 0.2387/undefined/undefined. The analysis including the non-responders yields the following for the High Dose (Resting/Waking/p-value): AUC 8.970e+05 ± 7.160e+05/5.411e+05 ± 3.662e+05/p=0.4897; RLU _max_ 1.970e+04 ± 1.710e+04/1.084e+04 ± 7.799e+03/p=0.4603; k 169.3542 ± 293.2279/4830333.3824 ± 8366382.7083/p=0.3739; t _1/2_ 8.5059 ± 8.1933/6.8643 ± 6.6622/p=0.8010; R ^2^ undefined/undefined/undefined.

## Description

Adeno-associated virus (AAV) vectors are the gold standard for delivering therapeutic genes for expression in the human body, but their adoption for gene therapy is hindered by the need for very large doses that increase risk of toxicity, with several high-dose trials resulting in patient deaths ("High-dose AAV gene therapy deaths," 2020), and that lead to large manufacturing cost, in some cases costing over $1 million per patient (Wang et al., 2019). Reducing the required AAV doses would be of immense benefit to patients, and a large community is invested in making gene therapy safe and affordable by engineering more effective AAVs and more efficient biomanufacturing processes.

Chronopharmacology aims to align the timing of medical treatments with endogenous circadian rhythms to maximize their benefits to patients (Hill et al., 2020). The efficacies of nearly 500 known drugs (Ballesta et al., 2017) and even surgeries (Montaigne et al., 2018) have been found to vary significantly with the time of day of administration (Ballesta et al., 2017; Kaur et al., 2013; Levi, 2001; Montaigne et al., 2018; Sancar et al., 2015; Tsimakouridze et al., 2015), but no studies have investigated the chronopharmacology of AAVs as a drug for therapeutic gene transfer. Immune system activity, which is a primary barrier to AAV therapy, varies dramatically over the circadian cycle, as do cells’ and animals’ susceptibility to infection by various pathogens and the effectiveness of vaccines for developing adaptive immune responses (Borrmann et al., 2021; Cervantes-Silva et al., 2022; Edgar et al., 2016; Feigin et al., 1972; Feigin et al., 1969; Kiessling et al., 2017; Liu et al., 2021; Scheiermann et al., 2013; Schmitz et al., 2022; Shackelford & Feigin, 1973). It has even been demonstrated that cells’ susceptibility to infection depends on cell-autonomous circadian clocks *in vitro* , independently of the immune system (Edgar et al., 2016). The similarities between AAV gene transfer and viral infection suggest that the mechanisms at play in these studies would likely have similar impacts on AAVs.

If AAV gene delivery is affected by circadian state similarly to viral infections, this could potentially be taken advantage of to reduce the prohibitive costs of gene therapy by requiring the use of fewer viral vectors per dose, may increase patient safety by dampening the toxic immune response to the AAVs, and could inhibit the adaptive immune response against AAVs that hinders repeated dosing. Thus, this study aimed to test the effect of the time of day (i.e., the resting or waking phase of the animal) of injection of an AAV gene therapy encoding a secreted luciferase reporter (El-Amouri et al., 2013) on the expression of the delivered transgene over several weeks in mice ( Figure 1A ).

Mice were given an intraperitoneal injection of AAV during either their Resting phase at Zeitgeber Time 6 (ZT6, at 12:00PM) or Waking phase (ZT18, at 12:00AM) ( Figure 1A ). It is common for AAV gene transfer to fail in some individuals due to pre-existing immunity (Vandamme et al., 2017), rendering their response dynamics incomparable to individuals that are transduced. Such “non-responder” individuals were identified using the criteria in the Statistical Analysis section and were excluded from the statistical analyses shown in Figure 1, while statistics including the non-responders can be found in the figure caption. The Low Dose experiment used n=4 mice per group and 2.5x10 ^9^ viral genomes/animal, with 1 non-responder in the Waking group. The Low Dose Resting group had remarkably higher transgenic *Gaussia* luciferase (GLuc) plasma levels throughout the 6-week measurement period than the Waking group ( Figure 1B ), measured in terms of relative light units (RLU). The area under the curve (AUC) were calculated for each time series of the Resting and Waking groups, and a two-tailed t test comparing the AUCs of the two groups yielded p=0.0037, allowing for rejection of the null hypothesis of no difference between the means, and indicating a statistically significant difference ( Figure 1C ). The experiment was repeated with a High Dose of 1.0x10 ^10^ viral genomes/animal and n=3 mice per group, with 1 non-responder in each group. Like the Low Dose experiment, the High Dose Resting responders had higher expression throughout the experiment than the High Dose Waking responders ( Figure 1B ). However, unlike the Low Dose trial, the Waking individuals in the High Dose group had high plasma levels of transgenic protein, and a two-tailed t test comparing the AUCs of the Waking and Resting groups yielded p=0.1311, lacking a statistically significant difference ( Figure 1C ). The Shapiro-Wilk test results for the AUCs of each group are the following: Resting Low Dose p=0.3210; Waking Low Dose p=1.0000; Resting High Dose p=1.0000; Waking High Dose p=1.0000, indicating normally distributed data.

To further characterize the dose responses, the data of each individual were fit with the model equation shown in Figure 1D, which was found previously to fit the RLU vs. time curves well for this AAV in mice (Burr et al., 2022) as it is a mechanistic model of the conversion of single-stranded AAV DNA to expressible, double-stranded DNA (Miao et al., 1998). RLU _max_ is the projected maximum steady state expression level, k is the rate constant, t _1/2_ is the time at which expression is half of RLU _max_ , and R ^2^ is the model fit. The fit parameters and R ^2^ values for the individuals are summarized in the table in Figure 1D as means ± standard deviations. P-values of t-tests comparing the Resting and Waking values for each dose level indicate statistically significant differences between Resting and Waking for all the metrics for the Low Dose (p<0.05) and none of the metrics for the High Dose.

Several known mechanisms may be contributing to the observed circadian effect. Phagocytes, especially in the liver, clear circulating nanoparticles at rates that vary with circadian phase (Hayashi et al., 2007; Kitchen et al., 2020; Labrecque & Cermakian, 2015; Scheiermann et al., 2013). Thus, the data in this report may be explained by a phagocyte-based threshold mechanism similar to one previously proposed to explain gold nanoparticle biodistribution (Ouyang et al., 2020). The given AAV dose would need to saturate the ability of the individual’s macrophages to clear the AAVs at the time of administration so that the excess AAVs can deliver genes to tissues. Literature suggests the Resting groups would have a lower ability to phagocytose AAVs, while the Waking groups would have higher ability. Thus, the Low Dose may not have been enough to overcome the Waking threshold but was enough to overcome Resting, while the High Dose was enough to overcome both Resting and Waking groups for the individuals that were responders.

Additionally, the immune response that destroys virally transduced cells acts against AAV activity (Martino & Markusic, 2020). As the effectiveness of multiple aspects of the immune system varies significantly with time of day (Scheiermann et al., 2013), this anti-viral activity likely varies as well. Intriguingly, the observed circadian effects could be independent of immune cells and instead result from circadian rhythms in the target cells (Husse et al., 2015), which impacts viral infection *in vitro* (Edgar et al., 2016) through mechanisms that are not yet understood.

These results imply that it may be optimal to inject human patients with AAV gene therapies during their resting phase (at night) to maximize transgene expression. Further studies are needed to narrow down the optimal timing of delivery—indeed, the optimal time to administer immune checkpoint inhibitors (Karaboue et al., 2024) and CAR-T cells (Tellinga et al., 2025) has been found to be shortly after the start of the waking phase, rather than the middle of the phases. Injecting AAVs when the immune system is less active could reduce toxic immune responses (Ertl, 2022) and reduce the adaptive immune response (Downton et al., 2020) that hinders future AAV gene delivery. Nighttime injections may be impractical, and not all patients share the same rhythms (Benloucif et al., 2008; Brown et al., 2008; Kasukawa et al., 2012; Upasham & Prasad, 2020), so identifying the mechanisms underlying the circadian effect could lead to complementary treatments to help improve AAV gene transfer. The circadian-dependence of AAVs could apply to other types of viral vectors or viral therapies (Marelli et al., 2018), or to nanoparticles in general (Gustafson et al., 2015), especially if phagocytosis is heavily involved. These findings also raise concern about the translatability of animal model results to humans—AAV therapies that work in mice when injected during the day may fail in humans when injected during the day due to mice being nocturnal and their circadian phases opposite of ours (Nelson et al., 2021). The knowledge of why AAVs are more effective when administered at certain times of day has the potential to cut costs of gene therapy by allowing the use of fewer viral vectors per dose, may increase safety by dampening the toxic immune response to the AAVs, and may inhibit the adaptive immune response against AAVs.

## Methods

*Animal husbandry*

Ten-week-old male C57BL/6J mice (Jackson Laboratories) were housed at up to four animals per cage and were allowed food and water ad libitum throughout the study. Mice were maintained in a light-dark cycle with lights on at 6:00AM and off at 6:00PM for at least one week before experiments.

*AAV vectors*

AAV2 vector encoding the secreted reporter protein *Gaussia* luciferase (GLuc) driven by an EF1α promoter was purchased from VectorBuilder. The AAV stock was thawed once to create aliquots at the concentrations used for injections in normal saline before being refrozen at -80 °C until use.

*AAV injections*

AAV aliquots were thawed immediately before injections. For each experiment, injections were first performed on the mice in the Resting groups at ZT6 (12:00PM). Twelve hours later, the Waking groups were injected at ZT18 (12:00AM). Each mouse was anesthetized with isoflurane before receiving an intraperitoneal injection of 100 μL of AAV containing 2.5x10 ^9^ viral genomes for the Low Dose experiment, or 1.0x10 ^10^ viral genomes for the High Dose.

*Transgenic protein measurement*

Each week following the injections, blood samples were collected by cutting the tail vein and collecting blood into heparin anticoagulant tubes. Within 1 hour after collection, the blood was assayed for relative GLuc concentration by mixing 100 μL of GLuc substrate solution consisting of 0.1% coelenterazine (NanoLight) in phosphate buffered saline with 20 μL of blood in a black-walled 96-well plate (Corning 3915) and immediately measuring the bioluminescent photon count (reported as relative light units, RLU) from the well over 10 seconds in a Varioskan plate reader.

**Statistical Analysis**

Time series of blood GLuc RLU are shown for each animal. The area under the curve (AUC) was calculated for each animal using trapezoidal numerical integration in MATLAB. Curves of the form RLU(t)=RLU _max_ *(1-exp(-k*t)) were previously found to fit the RLU vs. time curves well for this AAV in mice (Burr et al., 2022). This equation is mechanistic, modeling expression increase as the single-stranded DNA delivered by the AAV converts to double-stranded DNA with an exponential decay dynamic (Miao et al., 1998). The parameter RLU _max_ is the maximum, steady-state expression level that the mice reach once all single-stranded DNA is converted, and k defines the conversion rate. The metric t _1/2_ is the time at which the curve reaches half of RLU _max_ , and is calculated as t _1/2_ =-ln(1/2)/k. A curve was fit to the data for each mouse individually using the nlinfit function in MATLAB, and R ^2^ was computed as a goodness-of-fit. Three mice showed no rise in expression during the experiment and most likely are individuals with pre-existing immunity to AAVs that fail to be transduced—30-60% of humans have anti-AAV2 neutralizing antibodies, and even low levels of antibodies can entirely block transduction (Vandamme et al., 2017). The nlinfit method reported that the curve fit metric was insensitive to the given parameters for these mice, indicating that the model did not fit. We label these individuals “non-responders” and interpret them as a categorically different outcome and not fit for statistical comparison to the other mice. The subsequent statistical analyses were then performed twice: once excluding the non-responders, which is shown in the figure, and once including them, which is presented in the figure caption. AUCs for each group are plotted as means ± standard deviations, and the normality of the AUCs for each group was confirmed using the Shapiro-Wilk test with α=0.05. The Resting and Waking groups for each dose were compared using a two-tailed t test with α=0.05 in Excel (t-Test: Two-Sample Assuming Unequal Variances). The RLU _max_ , k, t _1/2_ , and R ^2^ values for each curve were likewise tested for normality using the Shapiro-Wilk test, and the Resting and Waking groups were compared using a t-test. Mean ± standard deviations and p-values are shown in the table in the figure.

## Reagents

**Table:** 

**STRAIN**	**GENOTYPE**	**AVAILABLE FROM**
C57BL/6J	Mus musculus	Purchased from Jackson Laboratories

**Table:** 

**AAV VECTOR TYPE**	**GENOTYPE**	**AVAILABLE FROM**
AAV2	AAV-EF1a-GLuc-WPRE	Purchased from VectorBuilder
